# Biomimetic strategies for the deputization of proteoglycan functions

**DOI:** 10.3389/fcell.2024.1391769

**Published:** 2024-08-06

**Authors:** Ibrahim F. Rehan, Asmaa Elnagar, František Zigo, Ahmed Sayed-Ahmed, Shuhei Yamada

**Affiliations:** ^1^ Department of Husbandry and Development of Animal Wealth, Faculty of Veterinary Medicine, Menoufia University, Shebin Alkom, Egypt; ^2^ Department of Pathobiochemistry, Faculty of Pharmacy, Meijo University, Nagoya, Aichi, Japan; ^3^ Department of Animal Nutrition and Husbandry, University of Veterinary Medicine and Pharmacy, Košice, Slovakia; ^4^ Department of Anatomy and Embryology, Faculty of Veterinary Medicine, Menoufia University, Shebin Alkom, Egypt

**Keywords:** proteoglycan, glycosaminoglycan, mimetic molecule, graft copolymer, therapeutic application

## Abstract

Proteoglycans (PGs), which have glycosaminoglycan chains attached to their protein cores, are essential for maintaining the morphology and function of healthy body tissues. Extracellular PGs perform various functions, classified into the following four categories: i) the modulation of tissue mechanical properties; ii) the regulation and protection of the extracellular matrix; iii) protein sequestration; and iv) the regulation of cell signaling. The depletion of PGs may significantly impair tissue function, encompassing compromised mechanical characteristics and unregulated inflammatory responses. Since PGs play critical roles in the function of healthy tissues and their synthesis is complex, the development of PG mimetic molecules that recapitulate PG functions for tissue engineering and therapeutic applications has attracted the interest of researchers for more than 20 years. These approaches have ranged from semisynthetic graft copolymers to recombinant PG domains produced by cells that have undergone genetic modifications. This review discusses some essential extracellular PG functions and approaches to mimicking these functions.

## 1 Introduction

Core proteins that are modified with a single or multiple glycosaminoglycan (GAG) chains are identified as a common class of biomolecules referred to as proteoglycans (PGs). GAGs are divided into two categories: sulfated and non-sulfated GAGs. PGs are the main *in vivo* source of sulfated GAGs containing heparin, heparan sulfate (HS), keratan sulfate (KS), chondroitin sulfate (CS), and dermatan sulfate (DS). Hyaluronan/hyaluronic acid (HA) is a non-sulfated GAG that is a main component of the extracellular matrix (ECM), but is not linked to a core protein (Schaefer and Schaefer, 2010).

Even though extracellular molecules make up the majority of PGs, they are also found inside cells or bound to cell membranes. Extracellular PGs may be further separated into three groups (Silbert, 2021). The first group consists of small leucine-rich PGs (SLRPs), which are primarily involved in osteogenesis and bone remodeling (Walimbe and Panitch, 2020). This group contains decorin and biglycan PGs, which consist of a single or multiple GAGs and a small protein core with a leucine-rich domain. SLRPs are divided into five distinct classes based on their genetic lineage and protein homology: three canonical SLRP classes, types I–III, and two non-canonical classes, types IV and V (Iozzo and Schaefer, 2015). While lumican is a member of SLRP class II and carries 2–4 KS chains, decorin and biglycan are members of SLRP class I and carry one and two CS/DS chains, respectively. SLRPs contain leucine-rich repeat units. Chondroadherin, nyctalopin, tsukushi, podocan, podocan-like 1, and testican-1, -2, and -3 are non-canonical members that share structural homology and have common functional characteristics despite not carrying GAG side chains (Iozzo and Schaefer, 2015; Gesteria et al., 2023). The second group is modular PGs, such as perlecan, the *N*-terminal domain I (amino acids 1–195) of which has three long 70- to 100-kDa GAG chains (Melrose, 2020). Perlecan is one of the HS-PGs, but transiently displays native CS chains during tissue morphogenesis. These CS chains are expressed by progenitor cell populations during tissue development (Schaefer and Schaefer, 2010; Iozzo and Schaefer, 2015; Sao and Risbud, 2024). Moreover, agrin is a large PG whose best-characterised role is in developing the neuromuscular junction during embryogenesis. It is named based on its involvement in the aggregation of acetylcholine receptors during synaptogenesis. In humans, this protein is encoded by the *AGRN* gene (Groffen et al., 1998; Kröger and Schröder, 2002; Rupp et al., 2015). There are three potential HS attachment sites within the primary structure of agrin, but it is thought that only two of these carry HS chains when the protein is expressed. Agrin may play an important role in the basement membrane of the microvasculature and synaptic plasticity. Also, agrin may be involved in blood-brain formation and/or function (Donahue et al., 1999; Wolburg et al., 2009) and it influences A*β* homeostasis (Rauch et al., 2011).

The third group is hyalectans, flexible HA-binding PGs that have four members: aggrecan, versican, neurocan, and brevican. Three distinct domains make up their core proteins: a HA-binding domain, a central domain containing GAG attachment sites, and a lectin-like domain. Depending on PGs, between three and 100 GAG chains are attached to the core proteins (Schaefer and Schaefer, 2010). Moreover, modular non-HA-binding PGs are primarily found in the basement membrane of tissues and have several forms (Schaefer and Schaefer, 2010). Extracellular PGs have attracted the most attention for the replication of physiological conditions encountered by cells in their natural tissue environment. Therefore, the main focus of this review will be extracellular PGs and attempts to mimic their functions.

## 2 Functions of PGs

The activities of PGs may be classified into four primary categories: a) the modulation of tissue mechanical properties; b) the regulation and protection of the ECM structure; c) protein sequestration; and d) the regulation of cell signaling. The structural composition of PGs is significantly affected by a number of factors, such as the size of the core protein and the type and number of conjugated GAGs. PGs have several physiological roles inside the human body. This section will concentrate on a few selected examples that are significant within the discipline of tissue engineering.

### 2.1 Modulation of tissue mechanical properties

GAGs exhibit a higher degree of hydrophilicity than the majority of other ECM constituents. This enhanced hydrophilicity is attributed to their sulfate and carboxylate groups, which provide negative charges that promote the absorption of water into tissue. Water absorption typically occurs in cartilage as well as embryonic developmental tissues (Arciniegas et al., 2004), and brain perineural nets (Ueno et al., 2018), particularly in regions abundant in the PG aggrecan (Knudson and Knudson, 2001; Kiani et al., 2002). The core protein of aggrecan is conjugated with approximately 60 KS and 100 CS chains. To immobilize HA chains in the ECM of cartilage, the core protein of the aggrecan molecule connects to HA via a link protein. This binding process results in the formation of large aggregates with a strong negative charge, which is important for enhancing the compressive stiffness of tissue as well as its ability to absorb water (Kiani et al., 2002).

Degenerative cartilage diseases, such as osteoarthritis (OA), may be caused by articular surface loss, which occurs as a result of progressive inflammation as well as excessive catabolic enzyme production, including aggrecanases, hyaluronidases, and matrix metalloproteases (MMPs) (Glyn-Jones et al., 2015). Close to the HA and aggrecan junction, aggrecanases enzymatically break the aggrecan core protein, releasing it from the network structure and facilitating its dispersion into synovial fluid (Lohmander et al., 1993). The first phase of OA is distinguished by the depletion of aggrecan, which reduced the ability of cartilage to maintain water. The promotion of cartilage loss is facilitated by an increase in the exposure of other ECM components to MMPs and hyaluronidases, and aggrecan loss promotes cartilage loss (Lohmander et al., 1993; Glyn-Jones et al., 2015). Previous studies extensively examined the biological and mechanical characteristics of aggrecan in cartilage (Kiani et al., 2002; Huang and Wu, 2008).

### 2.2 Regulation and protection of the ECM

The ECM mostly contains fibrillar collagen, which is involved in regulating the tissue structure such as skin, tendon, and mammary gland by providing cellular support (Sun, 2021; Melrose, 2024). The appropriate control of the production and structure of collagen fibrils is critical for ensuring the normal formation of the ECM and maintaining the functional properties of tissues. Decorin, biglycan, fibromodulin, and lumican are examples of SLRPs involved in the assembly of the ECM (Chen and Birk, 2013). Decorin is a PG that harbors a single GAG chain primarily composed of DS. In patients with Ehlers-Danlos syndrome who lack *Carbohydrate Sulfotransferase 14* (*Chst14*), this DS chain is substituted with CS. Decorin is a myokine that aids in the development of skeletal muscle in *Chst14*-deficient mice and is essential for the assembly of collagen fibrils (Miyake et al., 2010). Moreover, the binding of the core protein of SLRPs to collagen markedly affects the regulation of fibril development during collagen fibril growth (Douglas et al., 2006). The primary role of the decorin core protein is to facilitate the connection between PG and collagen. However, GAGs linked to the core protein also participate in charge-based interactions with surrounding collagen fibrils (Henninger et al., 2007). PGs and collagen fibril networks contribute to the properties and structure of the ECM, including mechanical stability and hydration (Robinson et al., 2005; Robinson et al., 2017).

Along with managing the organization as well as synthesis of collagen, SLRPs prevent its degradation by proteolysis. SLRPs safeguard collagen against destruction by shielding the external surface of collagen fibrils, thereby impeding the interaction between proteases and collagen (Geng et al., 2006). PGs other than SLRPs have been shown to protect against matrix proteolytic degradation. Aggrecan was found to impede the degradation of collagen II by inhibiting the access of MMPs to its significant bottlebrush structure and excluded volume (Pratta et al., 2003). In degenerative diseases, the degradation of PGs by proteases exposes collagen, rendering it susceptible to degradation (Ni et al., 2014). Further details on the effects of PGs on the matrix structure are provided in a number of reviews (Chen and Birk, 2013; Pang et al., 2019).

Elastins and fibronectin play a crucial role in the maturation and tissue specificity of the ECM. The binding of DS-PGs to plasma fibronectin (pFN) inhibits its interaction with multiple cell surface determinants (Laterra et al., 1983a; Laterra et al., 1983b; Woods et al., 1984). The binding of DS-PGs to pFN can interfere with the binding of pFN to the 140-kD glycoprotein receptor (Brown and Juliano, 1985; Cben et al., 1985; Giancotti et al., 1985; Pytela et al., 1985) or a possible second receptor for fibronectin (Aplin et al., 1981; Nagata et al., 1985; Urushihara and Yamada, 1986; Waite et al., 1987). Evidence for a conformational change upon GAG binding has been recently reported for human pFN (Tooney et al., 1983; Ostedund et al., 1985).

### 2.3 Sequestration of proteins

GAGs interact with not only matrix components, but also many different types of proteins, including morphogens, proteases, chemokines, and growth factors (GFs), to change their biological activity. These interactions are accomplished by the cationic domain of a protein, which consists of clusters of basic residues surrounded by one or two non-basic residues (Cardin and Weintraub, 1989; Fromm et al., 1997; Muñoz and Linhardt, 2004). However, the composition of the cationic domains of GAG-binding proteins varies, suggesting that specific sequences may not necessarily be required for interactions with GAGs. Alternatively, proteins may effectively bind to GAGs using a similar spatial structural motif, in which basic residues are close to one another in space, but not necessarily in the amino acid sequence (Zhang et al., 2019). In some case, the sulfation pattern of GAGs may also affect how proteins bind to them. For example, the primary binding interaction between the heparin-binding (HB) protein antithrombin III and heparin is mediated by a distinct sulfation pattern of pentasaccharides GlcNAc(6-*O*-sulfate)-GlcA-GlcN (2-*N*-sulfate, 3-*O*-sulfate, 6-*O*-sulfate)-IdoA (2-*O*-sulfate)-GlcN (2-*N*-sulfate, 3-*O*-sulfate, 6-*O*-sulfate (Yamada, 2019), where GlcNAc, GlcA, GlcN, and IdoA represent *N*-acetyl*-D*-glucosamine, *D*-glucuronic acid, *D*-glucosamine, and *L*-iduronic acid, respectively, observed on a heparin molecule subset (Lindahl et al., 1980; Atha et al., 1985; Merry et al., 2022). Moreover, the sequence required for the binding to basic fibroblast growth factor (bFGF) was a pentasaccharide containing *N*-sulfated GlcN residues and a 2-*O*-sulfated IdoA residue (Maccarana et al., 1993; Purushothaman et al., 2012). A previous study reported that the saccharide sequence of 6-*O*-sulfated oligosaccharide required for FGF signaling (Fernig D.G and Gallagher J.T., 1994; Lindahl et al., 1999; Gallagher, 2001; Turnbull et al., 2001; Kreuger et al., 2005; Sugaya et al., 2008).

Binding to GAGs immobilizes the protein, thus constraining and regulating its biological activity. The majority of membrane-bound PGs can also function as soluble autocrine or paracrine effectors as their extracellular domains, are enzymatically cleaved and released from the cell surface. In particular, the ectodomain shedding of syndecans, a major family of cell surface HS PGs, is an important posttranslational mechanism that modulates diverse pathophysiological processes. Syndecan shedding is a tightly controlled process that regulates the onset, progression, and resolution of various infectious and noninfectious inflammatory diseases (Nam and Park, 2012; García et al., 2016). HS-PGs bind to a number of GFs, such as members of the FGF (Ornitz, 2000), vascular endothelial growth factor (VEGF) as stated by Robinson et al., 2006, and platelet-derived growth factor (PDGF) families. HS-PG perlecan is ubiquitous throughout the body, but is mainly localized in the basement membrane (Whitelock et al., 2008). Perlecan plays a crucial role in establishing GF gradients to ensure proper tissue development by binding to GFs. This was confirmed using perlecan knockout models, in which the lack of perlecan led to tissue defects, including impairments in endochondral ossification and cardiovascular development (Arikawa-Hirasawa et al., 1999; Costell et al., 1999; Zoeller et al., 2008). The formation of tissues is disrupted by the lack of perlecan, while embryos experience premature mortality, thereby highlighting the crucial role of perlecan in maintaining optimal organ functionality. More details are provided in a number of reviews (Whitelock et al., 2008; Zoeller et al., 2008).

### 2.4 Regulation of cell signaling

PG-rich layers, such as the endothelial glycocalyx present in the vasculature, may prevent cell-cell interactions because of the anionic charge and steric hindrance of PGs (Reitsma et al., 2007; Tarbell and Cancel, 2016; Melrose, 2024). Consequently, no direct interactions may exist between vascular endothelial cells and circulating erythrocytes, leukocytes, and platelets in the blood. Upon injury to the glycocalyx from physical stress or the enzymatic degradation of GAGs, surface proteins on the endothelium, including selectins, VCAM, and ICAM, become visible (Lipowsky, 2012). Furthermore, the glycocalyx is involved in leukocyte recruitment, activation, arrest, and migration into the surrounding tissues. Effective artery repair necessitates this process. However, the inadequate restoration of the glycocalyx results in the unregulated activation of leukocytes, which induces uncontrolled inflammation at sites of injury (Reitsma et al., 2007). In addition to being structural proteins, PGs play a major role in signal transduction with regulatory functions in various cellular processes. Being mostly extracellular, they are upstream of many signaling cascades. They are capable of affecting intracellular phosphorylation events and modulating distinct pathways, including those driven by bone morphogenetic protein/transforming growth factor superfamily members, receptor tyrosine kinases, the insulin-like growth factor-I receptor, and Toll-like receptors. Mechanistic insights into the molecular and cellular functions of PGs have revealed both the sophistication of these regulatory proteins and the challenges that remain in uncovering the entirety of their biological functions (Schaefer and Schaefer, 2010).

## 3 Biomimetic strategies to replace PGs

**FIGURE 1:**
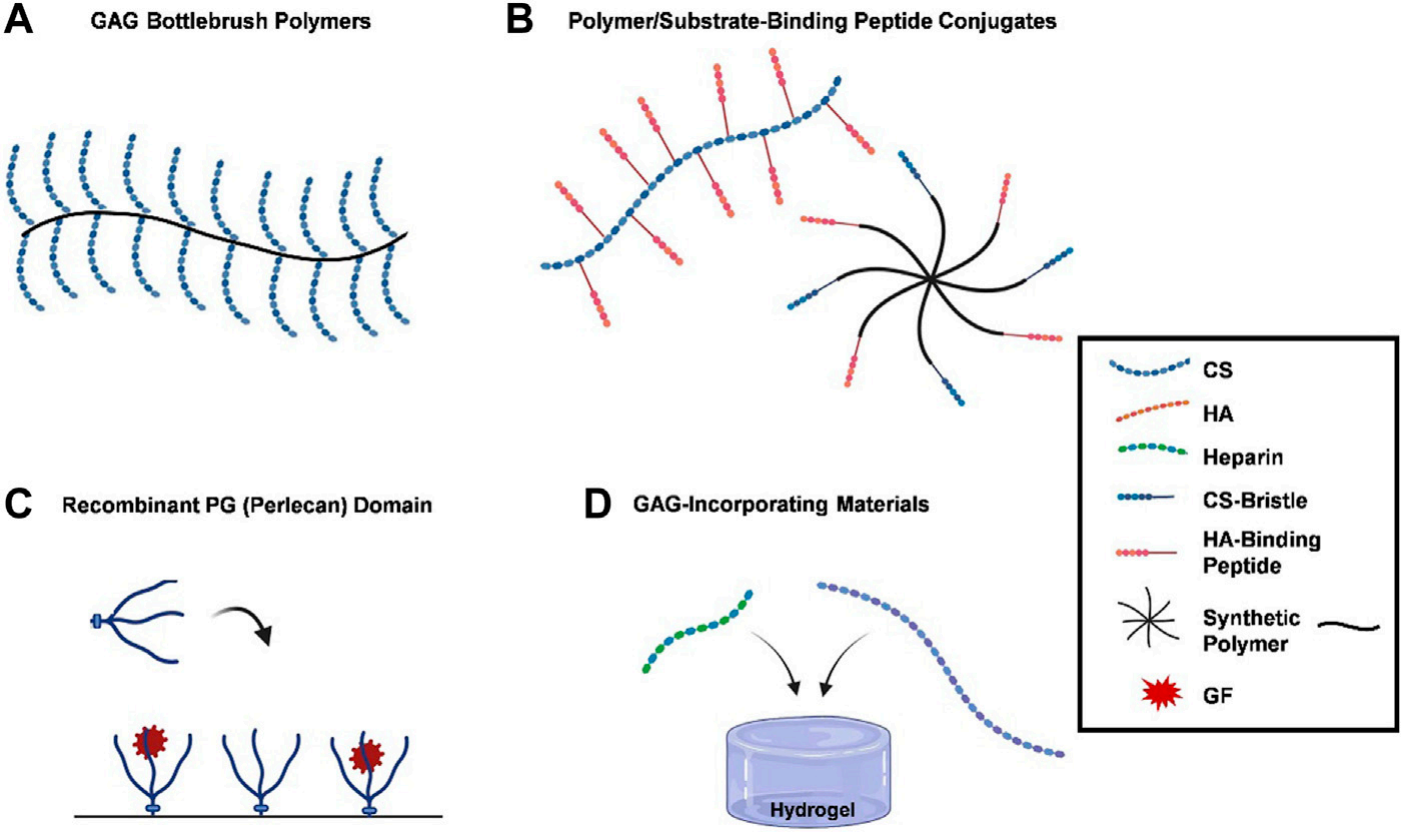
PG functions are recapitulated by biomimetic methods. **(A)** GAG bottlebrush polymers, graft copolymers are used to replicate the bottle brush-like structure of hyalectans like aggrecan, replicating their functions by grafting CS preparations to a synthetic poly (acryloyl chloride) backbone; **(B)** polymer/substrate-binding peptide conjugates, functional mimetics can be created by grafting substrate-binding peptides onto polymer matrices, which bind to HA or collagen, providing protection or imitating PGs organizational characteristics within the ECM; **(C)** recombinant PG (perlecan) domains, recombinant PG domains, such as terminal recombinant perlecan domains (rPlnDs), are used to replicate structural conformation of PGs while allowing for modification of their characteristics; and **(D)** GAG-incorporating materials, several research groups have opted for integrating GAGs into biomaterials instead of developing bespoke compounds, with heparin and CS being commonly used due to their commercial accessibility. A number of strategies have been developed to mimic the functions of PGs, ranging from the application of recombinant PG domains to the synthesis of PG mimetics. Blue dotted lines, red dotted lines, green dotted lines, a mixture of blue dotted and straight lines, a mixture of red dotted and straight lines, black lines, or red stars represent CS, HA, heparin, CS-bristle, HA-binding peptide, synthetic polymer, and GF, respectively.

Due to the significant contributions of PGs to the functions of healthy tissues, researchers have focused on developing methods that recapitulate these functions. Various techniques that simulate PG functions, including the use of recombinant PG domains and the development of semi-synthetic PG mimics, have been reported (see Figure 1). Four main goals to mimetic PG functions are highlighted herein.

### 3.1 GAG bottlebrush graft copolymers

**FIGURE 2:**
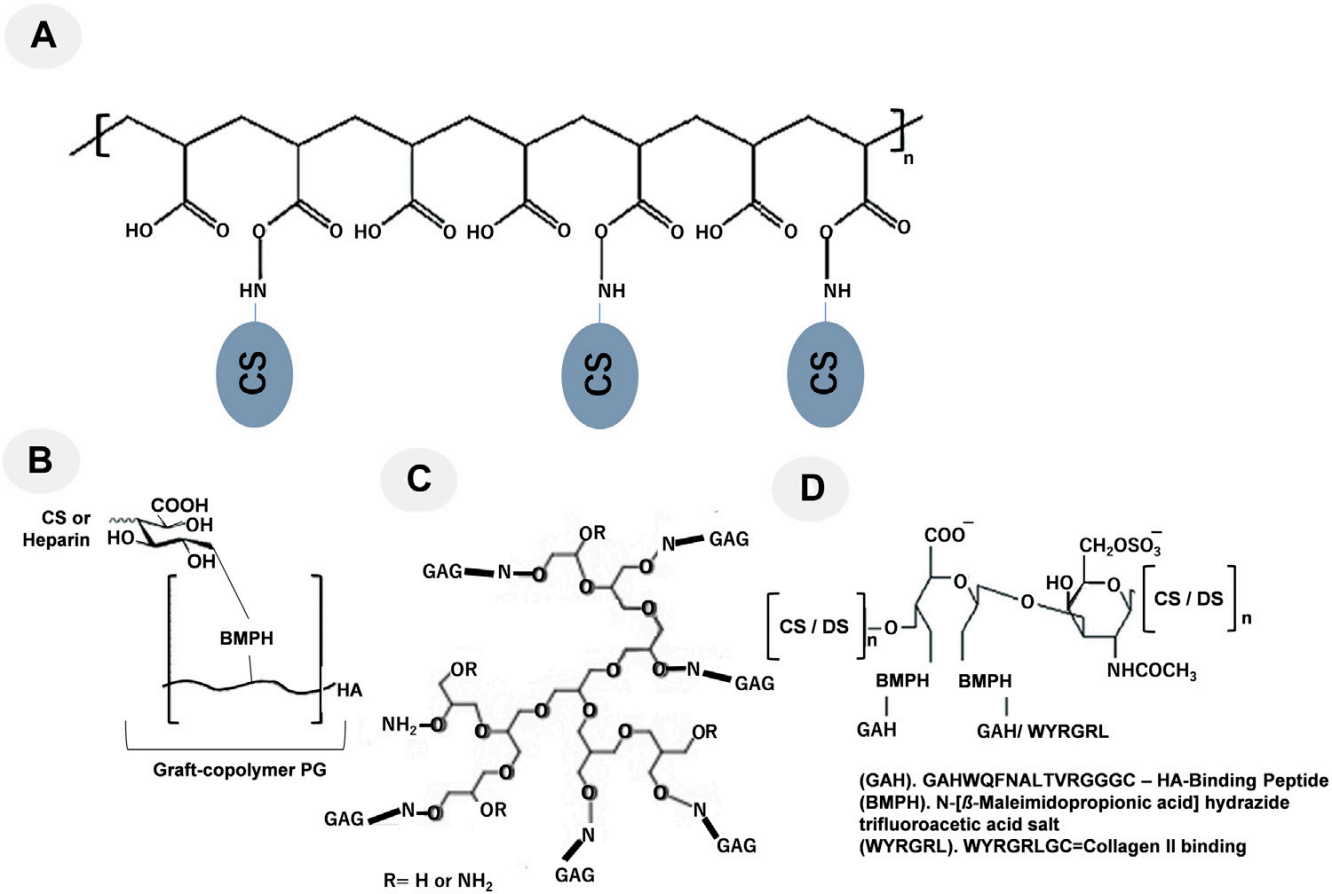
Representative graft-copolymer PG techniques are shown: **(A)** CS grafted to a poly (acryloyl chloride) backbone, modified from Prudnikova et al. (2018); **(B)** the HA backbone was grafted with heparin or CS, modified from Place et al. (2014a); **(C)** Grafting high molecular weight GAGs such as heparin and CS to hyperbranched synthetic cores like polyglycerol using oxime condensation, modified from Novoa-Carballal et al. (2018); and **(D)** the oxidized CS/DS backbone was conjugated to the linking compound BMPH and reacted with the synthetic HA-binding peptide GAHWQFNALTVRGGGC (referred to as GAH) or WYRGRLGC (collagen type II binding peptide, referred to as WYRGRL), modified from Bernhard and Panitch (2012), Sharma et al. (2016), respectively.

The use of graft copolymers to replicate the three-dimensional bottle brush-like structure of hyalectans, such as aggrecan, is one method for recapitulating the functions of PGs (Figure 1A). The types of biomimetic molecules are listed in Table 1. The grafting of CS preparations with a single terminal primary amine group to a synthetic poly (acryloyl chloride) backbone creates an aggrecan mimetic (Sarker et al., 2012; Prudnikova et al., 2017; Prudnikova et al., 2018; Clarke et al., 2024) (Figure 2A). Biomimetic PGs have been synthesized by combining 174 CS molecules (∼22 kDa) that were grafted onto a linear polymer of poly (acrylic acid) (PAA) backbone utilizing a reaction between a primary amine at the terminal end of CS and the acrylic acid groups in the PAA backbone (Prudnikova et al., 2018). This technique may be used to create small and large PG mimics with sizes ranging from a 10-kDa polyacrylate core with 7–8 CS chains attached (Prudnikova et al., 2017) to a 250-kDa core polymer with 60 CS chains attached (Prudnikova et al., 2018). The swelling of the molecule was found to be superior to that of aggrecan and unconjugated CS (Prudnikova et al., 2017). Moreover, much like SLRPs indicating the polymers behave like SLRPs with regards to controlling collagen fibril formation (Moorehead et al., 2019). Since negatively charged PAA did not affect the morphology of collagen fibrils, the CS structure rather than core proteins was considered to be important for controlling fibril formation. CS, either in its free-floating or PG form, interacts with collagen to regulate the kinetics of fibril formation and changes in diameters and band spacing (Prudnikova et al., 2018).

**TABLE 1:** The types of biomimetic molecules of PGs.

#	Type	Molecule/Peptide	Details	References
(A) Biomimetic Molecule				
1	GAG-PAA	BPG250	BPG250 contains of 174 CS molecule (∼22 kDa), which were grafted onto a polymer of PAA backbone utilizing a reaction between a primary amine at the terminal end of CS and the acrylic acid groups in the PAA backbone (Figure 2A)	Sharma (2012), Prudnikova et al. (2018), Phillips et al. (2019a)
		BPG10	BPG10 consists of a ∼10 kDa synthetic PAA core, decorated with ∼5–7 CS-GAG bristles (Figure 2A)	Kahle et al. (2022).
2	GAG-BMPH-HA	Heparin-BMPH-HA	Graft copolymers of heparin or CS with HA-BMPH (one polymer chain per thiol group in the HA-SH intermediate) (Figure 2B)	Sarkar et al. (2012), Place et al. (2014a), Pauly et al. (2017), Prudnikova et al. (2018)
		CS-BMPH-HA		
3	DS-PLL	PCNs	Prepared by a polymer-polymer pair reaction method and characterized for physicochemical properties. It is DS with poly-_L_-lysine (DS-PLL)	Zandi et al. (2020).
4	GAG-poly glycerol	Star-like PG	Grafting high molecular weight GAGs such as heparin or CS to hyperbranched synthetic cores like polyglycerol using oxime condensation (Figure 2C)	Novoa-Carballal et al. (2018).
5	CS-collagen	CSCL	Crosslinking CS onto a collagen-based scaffold.	Corradetti et al. (2016).
6	Collagen-HA-GAG	aCol-aHA-GAG	Chemically modified the collagen and HA are co-precipitated with GAGs. A bio-inspired nano-material recapitulating the composition, ultra-structure and function of the GAG-rich ECM is fabricated	Yang et al. (2023).
(B) Peptide-based Mimetic Strategies				
1	Peptide-CS/DS	GAH-oxidized CS	The peptide-glycan compounds prepared by this strategy include peptides that bind to ECM and are conjugated to a GAG backbone. These peptides, such as HA-binding peptides (GAH), are grafted to the oxidized CS/DS backbone (Figure 2D)	Bernhard and Panitch (2012), Sharma et al. (2013a), Zhang, et al. (2014), Lawrence et al. (2015), Sharma et al. (2016), Twitchell et al. (2020)
		DS-SILY	Collagen-binding peptides (SILY) conjugated to a DS backbone	Paderi and Panitch (2008), Scott et al. (2013)
		Lubricin mimic	Attaching type II collagen- and HA-binding peptides to a CS backbone	Lawrence et al. (2015).
2	Peptide-PEG	HA-binding peptide-PEG	HA-binding peptides are grafted onto PEG	Singh et al. (2014), Faust et al. (2018)

aCol-aHA; aminated collagen-aminated hyaluronan/hyaluronic acid-glycosaminoglycan; BMPH, *N-*[*β*-maleimidopropionic acid] hydrazide trifluoroacetic acid salt; BPGs, biomimetic proteoglycans; CS, chondroitin sulfate; CSCL, collagen-based scaffold; ECM, extracellular matrix; GAG, glycosaminoglycan; HA, hyaluronan/hyaluronic acid; PAA, polymer of poly(acrylic acid); PCM, pericellular matrix; PCN, polyelectrolyte complex nanoparticles; PEG, poly(ethylene glycol); PLL; poly-_L-_lysine.

One further method used to simulate aggrecan activity is a graft copolymer made of heparin or CS chains attached to the HA backbone through their reducing ends (Figure 2B) (Place et al., 2014b; Pauly et al., 2017). Sarkar et al. (2012) attempted to create graft copolymers in distinct compositions by utilizing four different ratios of either CS or heparin to HA-*N*-[*β*-maleimidopropionic acid] hydrazide trifluoroacetic acid salt (BMPH) (Prudnikova et al., 2018). Graft copolymers with 1:1, 1:3, 1:10, and 1:30 ratios were produced by mixing a stoichiometric amount of heparin or CS with HA-BMPH (one polymer chain per thiol group in the HA-SH intermediate). The 1:1 ratio showed the highest graft density. Only 1/30 of the stoichiometric amount of CS or heparin was made when the 1:30 ratio was used. After converting the carboxylic acid groups of HA to hydrazides, the graft polymer was synthesized by reductive amination. Smaller molecular weight polysaccharides decreased the modulus of the hydrogels, while larger molecular weight polysaccharides and graft copolymers increased the modulus (Pauly et al., 2017). The modulus of an agarose hydrogel was enhanced by the addition of the CS graft copolymer, and a similar effect was observed with the addition of free CS. These two additives were suggested to affect mechanical properties through different mechanisms. Free CS increased the modulus by chain entanglement, while the CS graft copolymer increased the modulus through osmotic pressure. However, the incorporation of the CS graft copolymer in agarose gels reduced cell viability, indicating the need for the further optimization of the mimetic. Although these mimetic strategies imitate the brush structure of hyalectan PGs by grafting GAGs onto a core polymer, they do not encompass the functions of the core protein (Pauly et al., 2017).

Brush PGs are intentionally engineered with a reduced binding affinity towards HA in order to promote tissue localization and minimize unintended molecular diffusion (Pratta et al., 2003; Pauly et al., 2017; Moorehead et al., 2019). Within the realm of therapeutic interventions, this process may give rise to a gradual increase in the dosages of hyalectan PGs required to maintain optimal performance (Maneiro et al., 2004). However, the advantages of increased resistance to proteolytic breakdown, which may be accomplished by replacing the protein core with an alternative polymer, may outweigh the disadvantages that arise from the loss of protein core functions. Further research is required to obtain a more accurate assessment of the equilibrium between functional imitation and effective therapeutics (Pratta et al., 2003; Pauly et al., 2017; Moorehead et al., 2019; Murphy et al., 2019; Hwang et al., 2020; Lanzi et al., 2020; Rizzo et al., 2022; Yao et al., 2022; Zhang et al., 2022; Abdelfadiel et al., 2023; Heide et al., 2023; Kardeby et al., 2023; Xie et al., 2023; Zhang et al., 2024).

### 3.2 Peptide-based mimetic strategies

An alternative approach to fabricating functional mimetics comprises the incorporation of substrate-binding peptides onto polymer matrices (Figure 1B). These peptides have a propensity to bind to HA or collagen, resembling the binding domains of hyalectans or SLRPs. It is possible to offer protection or imitate the organizational characteristics of PGs within the ECM after grafting these peptides onto polymers.

The peptide-glycan compounds prepared by this strategy include peptides that bind to the ECM and are conjugated to a GAG backbone (Petrovic, 2024). These peptides, such as HA-binding peptides (GAH), are grafted to the oxidized CS backbone (Figure 2C). Although PGs often have a single ECM-binding domain, mimetics were generated by grafting 5–15 peptides onto the GAG backbone, taking advantage of avidity to enhance target binding. This methodology utilizes avidity as a means to augment binding affinity towards the desired target. The use of an aggrecan mimetic, a CS backbone grafted with HA-binding peptides, demonstrated that this strategy supported the protection of the ECM against its proteolytic degradation (Bernhard and Panitch, 2012; Sharma et al., 2013b; Zhang, et al., 2014; Sharma et al., 2016). A lubricin mimetic was created by combining HA-binding peptides with a CS backbone (Lawrence et al., 2015; Twitchell et al., 2020), and it was placed on the surface of articular cartilage, which lowered the coefficient of friction. Previous studies demonstrated that a synthetic compound resembling decorin offered a safeguard against the breakdown of proteins by enzymes and controlled the formation of collagen fibers. This is achieved by the compound’s interaction with collagen via specific peptide sequences that are connected to the decorin DS backbone (Paderi et al., 2009; Stuart et al., 2011).

This approach effectively restored the glycocalyx after vascular endothelial denudation by the collagen-binding decorin mimetic (Scott et al., 2013), called DS-SILY, which markedly inhibited platelet activation by covering exposed collagen, thereby aiding in the suppression of vascular intimal hyperplasia. Furthermore, DS grafted with selectin-binding peptides was utilized to protect an inflamed endothelium (Dehghani et al., 2020). Both mimics were located in the damaged vessel to recapitulate the GAG barrier by preventing vessel interactions with neutrophils and circulating platelets. Since peptides that bind to HA have been shown to reduce friction on the surface of articular cartilage (Singh et al., 2014; Lawrence et al., 2015), a lubricin mimic was developed by grafting HA-binding peptides onto poly (ethylene glycol) (PEG) (Singh et al., 2014; Faust et al., 2018; Zhou et al., 2022). The PEG-based lubricin mimic was administered through an intravenous injection into an OA animal. The molecule subsequently bound to HA, causing HA to accumulate in the cartilage and reduce joint friction (Singh et al., 2014). The application of this specific molecule in a murine model of OA suppressed disease progression, which appeared to be attributed to the higher concentration of HA on the cartilage surface (Faust et al., 2018).

Moreover, as shown in Figure 2C, grafting high molecular weight GAGs such as heparin and CS to hyperbranched synthetic cores like polyglycerol using oxime condensation (Novoa-Carballal et al., 2018).

Although the structures of these molecules differ from those of the PGs they mimic, they have the functions of the binding domains on the core proteins of PGs, leading to their local retention in order to extend the duration of their therapeutic effect. Nevertheless, it is important to note that the ability of these synthetic analogs to fully replicate the functions of PGs may be limited due to their smaller dimensions than PGs and the utilization of their peptides may render them more vulnerable to destruction by peptidases (Figure 2D). Moreover, in Table 2, we have summarized the therapeutic applications of PGs, modified from recent publications (Walimbe and Panitch, 2020; Rizzo et al., 2022; Zhang et al., 2022; Yao et al., 2022; Abdelfadiel et al., 2023; Heide et al., 2023; Kardeby et al., 2023; Xie et al., 2023; Zhang et al., 2024).

**TABLE 2:** Summary of PGs and their therapeutic applications.

PGs	Predominant GAG	Therapeutic application	Function	References
Glypican 1–6	HS	Ischemic wound healing	Developmental morphogenesis	Monteforte et al. (2016).
		Suppressing metastasis in gastric cancer		Han et al. (2016).
Syndecan 1–4	HS	Diabetic wound healing	Cell adhesion; binding to FGF and other growth factors	Das et al. (2016a), Das et al. (2016b), Das et al. (2016c)
Aggrecan	CS, KS	Osteoarthritis	Mechanical support; forms large aggregates with HA	Bernhard and Panitch (2012), Sarkar et al. (2012), Sharma et al. (2013a), Place et al. (2014a), Place et al. (2014b), Sharma et al. (2016), Prudnikova et al. (2017), Prudnikova et al. (2018), Phillips et al. (2019b)
Decorin	DS	Macular degeneration, diabetic retinopathy, diabetic macular edema	TGF-ß binding and Fibrillogenesis	Devore et al. (2010).
		Corneal wound healing		Grisanti et al. (2005), Hill et al. (2018)
		Anti-scarring		Stuart et al. (2011), Ahmed et al. (2014), Wang et al. (2016)
		Oncosupression		Yang et al. (2015), Wang et al. (2016), Oh et al. (2017a), Liu et al. (2017)
		Abdominal aortic aneurysm		Shen et al. (2017).
		Vascular neointimal hyperplasia		Paderi et al. (2011), Scott and Panitch (2014), Scott et al. (2017)
Lumican	KS	Corneal wound healing	Cell adhesion	Chakravarti (2002), Gesteira et al. (2017)
		Bacterial lung infections		Shao et al. (2012), Shao et al. (2013)
		Scarring		Yeh et al. (2005), Liu et al. (2013), Yamanaka et al. (2013), Zhao et al. (2016)
		Melanoma		Zeltz et al. (2009), Pietraszek et al. (2013)
Biglycan	CS	Duchenne muscular dystrophy	Cell adhesion	Amenta et al. (2011), Ito et al. (2017), Fallon and McNally (2018)
Fibromodulin	KS	Diabetic wounds and neuropathy	Cell adhesion and fibrillogenesis	Jian et al. (2013), Zheng et al. (2014), Jazi et al. (2016)
		Neointimal hyperplasia		Ranjzad et al. (2009).
		Bone regeneration		Zheng et al. (2012), Li et al. (2016)
		Tendon healing		Delalande et al. (2015).
		Breast cancer metastasis		Dawoody et al. (2017).
Lubricin	None	Osteoarthritis	Lubrication	Ludwig et al. (2012), Wathier et al. (2013), Lawrence et al. (2015), Iqbal et al. (2016), Larson et al. (2016), Lakin et al. (2019)
		Ocular applications, dry eye		Oh et al. (2017b), Lambiase et al. (2017), Samsom et al. (2018)
Perlecan	HS	Cartilage regeneration	Stability of basement membranes and providing filtration barrier	French et al. (2002), Yang et al. (2006), Jha et al. (2009), Srinivasan et al. (2012)
		Ischemic wound healing		Aviezer et al. (1994), Zoeller et al. (2009)
		Neurovascular dysfunction		Clarke et al. (2012), Parham et al. (2016)
		Stroke and vascular dementia		Lee et al. (2011), Marcelo and Bix (2015)
		Neointimal hyperplasia		Rnjak-Kovacina et al. (2016).

CS, chondroitin sulfate; DS, dermatan sulfate; GAG, glycosaminoglycan; HS, heparan sulfate; KS, keratan sulfate; PG, proteoglycan.

### 3.3 Recombinant protein domains

Recombinant PG domains represent an alternative approach for replicating the structural conformation of PGs, while it retains the flexibility to change their characteristics (Figure 1C). While pioneering researchers have focused on the roles of GAGs and PGs for years, in the last two decades the staggering potential of PGs to modulate tissue environments has been more broadly appreciated. Their multifunctional biological processes, in particular, their ability to bind and sequester GFs and interact with various ECM molecules and influence cellular signaling events, makes them extremely attractive drug conjugates for multiple disease indications. Table 2 summarizes common PGs and their therapeutic applications. Clinical translation of these molecules, however, remains a challenge. Due to the advent of recombinant technology, adenoviral and non-viral gene transfers are attractive alternatives to purify native PGs, a task that is considered extremely difficult and time intensive. However, while recombinant technology can synthesize core proteins of PGs fairly consistently, their post-translational GAG chain modifications remain a challenge. Some GAG chain structures require enzymes in the Golgi apparatus only found in multicellular eukaryotes, but absent in single celled organisms used to synthesize recombinant PGs. Effectively conveying the mechanism of action of these drugs also remains a significant challenge, due to the diverse processes with which these molecules interact.

Terminal recombinant perlecan domains (rPlnDs) with the native attachment of HS chains are commonly utilized for this purpose (Whitelock et al., 2008). These rPlnDs may be produced by transfecting mammalian cells, leading to the production of the core protein and the HS chains to be connected thereafter (Chiu et al., 2016; Hubka et al., 2019). To regulate the interaction, exhibition, and liberation of GFs that have an affinity for heparin, this methodology was employed to fix rPlnDs onto diverse surfaces (Knox and Merry, 2002; Marneros and Olsen, 2005; Whitelock et al., 2008). The Farach-Carson group conducted an experiment wherein they utilized rPlnDI to demonstrate the prolonged liberation of BMP-2 from a scaffold made of poly (ε-caprolactone) (PCL) through the process of electrospinning (Chiu et al., 2016). The achievement of this task involved the covalent attachment of rPlnDI to scaffold fibers before the addition of BMP-2 (DeCarlo et al., 2012; Chiu et al., 2016). The immobilization of rPnlDI resulted in the greater loading of BMP-2 within the scaffold than with a control PCL scaffold. Additionally, the immobilized scaffold exhibited the prolonged release of BMP-2 over an extended duration. The research team also employed a custom-designed microfluidic device fabricated by 3D printing technology to generate varying concentrations of rPlnDI. These concentration gradients were subsequently utilized to induce corresponding gradients of FGF-2 within the hydrogel matrix (Hubka et al., 2019). In contrast to hydrogels with a uniform distribution of FGF-2, the presence of a gradient of FGF-2 facilitated enhanced cell migration. Furthermore, rPnlDI was successfully attached to microparticles to facilitate the regulated release of GFs, such as BMP-2. The release kinetics of this technique were superior to the distribution of GF by a free delivery system (Jha et al., 2009).

Moreover, the anabolic process of chondrocyte aggrecan recombination is very dynamic during the development of OA. The domains of aggrecan protein contain several cutting sites susceptible to MMPs, ADAMTS, and other enzymes (Santamaria and Yamamoto, 2020). Aggrecan plays an important role in mediating chondrocyte-chondrocyte and chondrocyte-matrix interactions through its ability to bind HA (Kiani et al., 2001; Kiani et al., 2002; Miwa et al., 2006). Given that the biological function of aggrecan depends on fixing charges in the cartilage ECM, tethering the PG is vital. The lectican PGs are incorporated into the ECM through specific interactions with HA and other ECM components. These interactions are mediated through their globular domains. Recent findings of missense mutations in the aggrecan genes in patients with different skeletal disorders emphasize the importance of the globular domains of aggrecan (Aspberg, 2012).

In the central and peripheral nervous system, versican is expressed by glial cells and is implicated in the regulation of cell adhesion, migration, pattern formation, and regeneration. Recombinant versican specifically binds HA and does not bind to heparin or CS. The transfected fibroblasts make a 78-kDa truncated form of versican that also binds HA but not the related polysaccharides, showing that the HA-binding activity resides at the *N*-terminus of versican. The binding of versican to HA is substrate-concentration -dependent and time-dependent, and can be competed HA with unlabeled versican. Versican interacts with tenascin-R via its *C*-type lectin domain (Asperg et al., 1995). This interaction has been shown to contribute to the formation of perineuronal nets around neuronal cells towards the end latter stages of brain development, a process that is thought to inhibit synaptic plasticity (Carulli et al., 2006; Anlar and Gunel-Ozcan, 2012). Through the same lectin domain versican can also interact with fibulin-1, fibulin-2 and fibrillin-1 (Asperg et al., 1999; Olin et al., 2001; Isogai et al., 2002). As these proteins are associated with elastic microfibrils, it is hypothesized that these complexes may also play a role in regulating elastogenesis (Wight, 2002; Bashur and Ramamurthi, 2012).

Several studies have examined the recombinant domain V of the *C* terminus of perlecan. Endorepellin has been characterized as a potent anti-angiogenic molecule with a stated form covering the domain from Glu3687 to Ser4391. However, a large form known as rPlnDV, which contains the amino acids Leu3626 to Ser4391, has been shown to promote angiogenesis (Rnjak-Kovacina et al., 2017). Despite the two reported recombinant domains sharing the integrin α2*β*1-binding site, the latter has an HS or CS chain attached, whereas endorepellin has no GAG chains, indicating the significance of the GAG chain in the signal transduction of angiogenic GFs (Rnjak-Kovacina et al., 2017). Lin et al. (2020) examined whether the mimetic of the large GAG-bound molecule promotes angiogenesis by potentiating available GFs both in their soluble and immobilized forms to silk fibroin scaffolds. Following the removal of GAG chains from rPlnDV, cells did not produce proangiogenic signals, indicating that GAG chains are necessary for GF potentiation.

Concerning the potential of an immune response to the protein core of recombinant PGs. For instance, syndecan, perlecan, biglycan, decorin, lumican, and especially versican, provide fine control of innate immunity by binding to a number of recognized immunoregulatory molecules—chemokines, cytokines, GFs, and MMPs—mostly mediated by ionic charge interactions, as extensively discussed elsewhere (Schmidtchen et al., 2001; Venkatesan et al., 2002; Gill et al., 2010; Kehlet et al., 2017; Kang et al., 2018). Therefore, innate immune signaling by PGs indicates the implications and direction to define great progress in this area of ECM biology. Since recombinant PG domains comprise both GAG chains and parts of the core protein, the molecules produced are similar to their respective PGs in terms of both form and function. This is the most intricate and costly mimetic approach because it requires genetically modified cells to synthesize molecules.

### 3.4 GAG-functionalized materials

To recapitulate the GF-binding ability of PGs, many groups have opted to use unmodified GAGs as biomaterials, rather than synthesizing specialized molecules. Heparin is commercially available and which is generally derived from porcine intestine (Kim et al., 2018; Levinson et al., 2019; Hettiaratchi et al., 2020; Subbiah et al., 2020; Wang et al., 2020), as well as CS preparations (Karumbaiah et al., 2015; Betancur et al., 2017; Birdwhistell et al., 2018), respectively, are commonly employed in this context due to their widespread commercial accessibility.

A modular system of biohybrid hydrogels based on covalently cross-linked heparin and star-shaped poly (ethylene glycols) (star-PEG) in which network characteristics can be gradually varied while heparin contents remain constant (Freudenberg et al., 2009). Moreover, Levinson et al. (2019) established a hydrogel composed of HA and heparin, which was a carrier for TGF-*β*1 and chondrocytes (Figure 1D). This study described the utilization of two hydrogels, one of which incorporated HA crosslinked with heparin, while the other hydrogel consisted of heparin without any cross-linking. Both gels were formulated with TGF-*β*. The gel with cross-linked heparin showed a sustained release profile, whereas the gel with uncross-linked heparin exhibited a bigger initial burst release of GF. The production of collagen II and GAG was greater by encapsulated cells than by those cultured in medium supplemented with TGF-*β* (Levinson et al., 2019). In an investigation of the treatment of ischemic wounds, Kim et al. (2018) successfully trapped VEGF using a gelatin cryogel functionalized with heparin. Since the level of heparin integrated was elevated, a larger amount of VEGF was preserved over time. In a rat ischemia hind limb model, the construct was implanted with NIH-3T3 fibroblasts and VEGF-laden cryogels. The induction of angiogenesis was weaker by gels loaded with only VEGF or cells alone than by a gelatin cryogel functionalized with heparin, suggesting that sequestered GFs by sulfated GAGs have an impact on tissue engineering. Other research groups reported similar findings, namely, the enhancement of tissue strength was achieved through the sequestration of GFs by their interaction with sulfated GAGs (Yu et al., 2000; David et al., 2014; Diana et al., 2017; Daniel et al., 2019; Bryce et al., 2023). The spatial patterning of GFs may be achieved by incorporating GAGs using techniques such as controlled deposition (Oh et al., 2018) and 3D printing (Wang et al., 2020). Moreover, an injectable clinical biomaterial must meet marketing, regulatory, and financial constraints to provide affordable products that can be approved, deployed to the clinic, and used by physicians. Many HA-derived hydrogels can deliver cells and therapeutic agents for tissue repair and regeneration (Burdick and Prestwich, 2011).

Sulfated HA, especially high-sulfated HA (hs-HA), blocks *Heparanase* (*Hpse*)-mediated enzymatic actions and cellular functions, that is, invasion into the surrounding ECM and *Hpse*-mediated upregulation of the chemokine CCL2 released from colon-26 carcinoma cells. Therefore, sulfated HA is potentially considered as anti-metastatic and anti-inflammatory agent via inhibition of *Hpse* functions (Shi et al., 2022). The sulfation involves the *C*-6 and *C*-4 positions of glucosamine and the *C*-2 and *C*-3 positions of glucuronic acid. Moreover, sulfated HA has also been employed in the sequestration of GFs. The advantage of this strategy is that the degree of sulfation may be controlled, thereby lowering the chance of heparin intrinsic antithrombotic activity (Feng et al., 2017; Thönes et al., 2019).

The sequestration and controlled release or presentation of GFs, such as FGF-2 (Karumbaiah et al., 2015), VEGF (Kim et al., 2018; Subbiah et al., 2020), transforming growth factor-beta (TGF-*β*) (Birdwhistell et al., 2018; Levinson et al., 2019), BMP-2 (Hettiaratchi et al., 2020; Subbiah et al., 2020), and nerve growth factor (Oh et al., 2018), have been achieved by integrating these proteins into materials.

## 4 Conclusion and prospective

Efforts to mimic PGs in the cellular environment have mainly focused on ECM PGs and membrane-anchored PGs. PG mimetic strategies have varied, with most incorporating GAGs. However, due to the complexity of PG structures, no mimic fully replicates their functions. Bottlebrush copolymers, which lack HA-binding sites, may regulate swelling behavior, but do not fully replicate their structural characteristics. Recombinant PG domains can accurately replicate native PGs; nevertheless, PG biosynthesis and genetic engineering pose challenges to the scaling up of recombinant technology. Due to their greater degree of control over synthesis and optimization, cost effectiveness, and ability to impart bioactivity comparable to that of their native equivalents, PG mimetics are becoming more common. However, there are difficult challenges with synthetic methods when it comes to producing quantities that are beneficial to the market. The next-generation of PG therapeutics to target a wide range of disorders is being developed by scientists for the synthetic GAG and sequencing, as well as knowledge of the kinetics of PG binding interactions with growth factors. To improve binding interactions, important characteristics of GAG length and sulfation along with interactions with core proteins are being changed. Interest in concentrating on the precise and regulated release of these PGs is also growing. For improved treatments, engineering PGs to sequester and regulate GF release is being investigated. To develop targeted and effective treatments, strategies for targeting particular tissues are being investigated. One such strategy involves taking use of core proteins” capacity to attach to collagen or HA. In order to manage and adjust particular sulfation patterns, binding potential, and specificity of mimetics to build novel ways of influencing disease states, synthetic approaches are being used to get around the variety of native PGs. Ultimately, researchers are developing new mimetics that imitate not just the structure but also the functionality of PGs. The links between PG structure and function are still mostly unknown. However, early preclinical research has demonstrated the potential of PG therapies to lead the way in novel therapeutic approaches and advancements across a range of disease indications, including diabetes, cancer, osteoarthritis, wound healing, and hypertrophic scarring. Overall, the discovery of PG- and GAG-based therapeutics is bringing the importance of the extracellular matrix (ECM) for tissue health and cell function back into focus and creating new opportunities for the creation of bioinspired and targeted medication classes.

## Figures and Tables

**Table:** 

**b-PLA**	biotin end group poly (lactic acid)
**BPGs**	biomimetic proteoglycans
**bFGF**	basic fibroblast growth factor
**BMP-2**	bone morphogenic protein 2
**BMPH**	N-[β-maleimidopropionic acid] hydrazide trifluoroacetic acid salt
**CS**	chondroitin sulfate
**CSCL**	collagen-based scaffold
**Chst14**	Carbohydrate Sulfotransferase 14
**DS**	dermatan sulfate
**ECM**	extracellular matrix
**FGF**	fibroblast growth factor
**GAG**	glycosaminoglycan
**GF**	growth factor
**HA**	hyaluronan/hyaluronic acid
**HB**	heparin-binding
**HS**	heparan sulfate
**KS**	keratan sulfate
**MMP**	matrix metalloprotease
**OA**	osteoarthritis
**PAA**	polymer of poly (acrylic acid)
**PCL**	poly (ε-caprolactone)
**PCM**	pericellular matrix
**PCN**	polyelectrolyte complex nanoparticles
**PDGF**	platelet-derived growth factor
**PDMAEMA**	poly (2-dimethylaminoethyl methacrylate)
**PEG**	poly (ethylene glycol)
**PG**	proteoglycan
**PMPC**	poly (2-methacryloyl phosphorylcholine)
**PMVK**	poly (methyl vinyl ketone)
**rPlnD**	recombinant perlecan domain
**SLRP**	small leucine-rich PG
**TGF-β**	transforming growth factor-beta
**VEGF**	vascular endothelial growth factor.
